# Has the cat got your tongue, or is something obstructing your throat? A review of imaging of ingested and aspirated foreign bodies in the paediatric population

**DOI:** 10.1007/s00247-024-06068-3

**Published:** 2024-10-18

**Authors:** Ola Kvist, Juan Pablo Garcia

**Affiliations:** 1 Department of Women’s and Children’s Health, Karolinska Institute, Stockholm, Sweden; 2 Department of Radiology, Columbia University Medical Center, New York, NY USA

**Keywords:** Child, Computed tomography, Fluoroscopy, Foreign bodies, Radiography, Ultrasonography

## Abstract

Children frequently swallow or inhale foreign objects, a situation that can be life-threatening. Radiographic imaging plays a lead role in the early identification and location of inhaled or swallowed objects is essential. Promptly identifying and locating inhaled or swallowed objects are essential, as some items require immediate removal. For example, button batteries in the throat can cause grave harm; magnets can attract each other through the gut and cause perforations; and other objects can obstruct the airway or intestinal tract. Radiologists must understand how these objects appear in images to assist doctors in treating patients effectively. Recognising signs of inhaled objects on radiographs is also crucial, as symptoms may not always be clear, and many inhaled objects are not visible on radiographs. Radiographs are the primary means of checking for swallowed or inhaled objects, although other tests like fluoroscopy and computed tomography may be used in complex cases. Doctors working with children should be acquainted with the appearance of these common objects on images and their clinical importance.

**Graphical Abstract:**

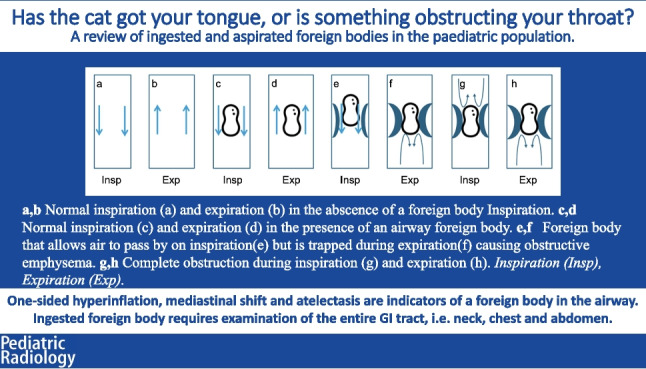

## Introduction

Ingested and aspirated foreign bodies, both organic and non-organic, remain a common cause of morbidity and mortality in children aged 1 to 3 years [1–3]. Early detection and treatment are vital to minimise these consequences. The approach to detect foreign bodies can vary depending on the suspected location of the foreign body and the available imaging modalities. The size, shape and material of the foreign body may also influence the type and urgency of the intervention. Based on the location of a foreign object in the body, as determined by a prior clinical assessment, frontal and lateral radiographs of the neck, thorax or abdomen may be performed. Supplemental imaging, such as oblique projections or additional expiratory views of the thorax, can be employed to confirm the diagnosis, particularly when an endobronchial body is suspected.

The purpose of this review is to explore current strategies for the detection of foreign bodies in the airway and the gastrointestinal tract, alongside various factors that influence the monitoring and intervention of a patient.

## Foreign bodies in the airway

The role of radiology is to determine the presence and location of a foreign body in the airway, so that it may be extracted. The role of radiology is to ascertain the presence and location of a foreign body in the airway, so that it may be removed. The location of the foreign body influences the urgency of intervention, from immediate (larynx and trachea) to lower priority when it is more distal in the segmental, subsegmental bronchi. Bronchoscopy is considered the gold standard technique for extracting foreign bodies from the airways of children. However, this technique requires general anaesthesia and carries a potential risk of complication. It should therefore only be utilised once a foreign body is suspected. A radiological challenge is that the majority of aspirated or inhaled foreign bodies comprise organic material and are thus radiolucent. Consequently, a negative radiograph does not exclude the presence of a foreign body in the airways.

Most children experience witnessed aspiration of a foreign body, accompanied by choking, before arrival at the accident and emergency department [4]. A history of choking is the most reliable indicator of all clinical symptoms, with a reported sensitivity of 97% and specificity of 63% [5]. An unknown history of foreign body aspiration can lead to delayed presentation to the emergency department, as well as an increased risk of the patient being misdiagnosed as having recurrent respiratory infections or asthma [4]. The most conventional workflow for a child with known aspiration or cough symptoms is a chest radiograph, as it is economical and widely available.

## Radiograph

**Fig. 1:**
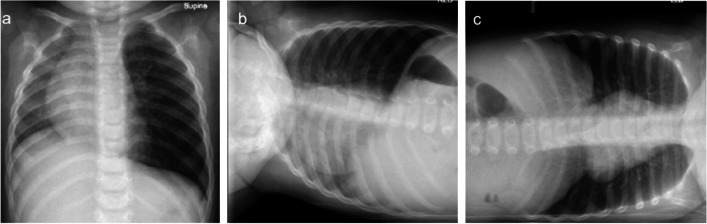
Radiographs of a 2-year-old boy who has choked whilst consuming carrots. **a** The anteroposterior (AP) view shows an expanded and hyperlucent left lung with a mediastinal shift to the right hemithorax. **b** Right lateral decubitus view shows a reduced aeration in the right lung, i.e. no sign of air trapping. **c** The left lateral decubitus view demonstrates the aeration in the left lung is maintained and the lung volume is almost constant to the contralateral side as well as unchanged compared to the AP view. The findings are conclusive with air trapping indicating a foreign body in the left main bronchus

**Fig. 2:**
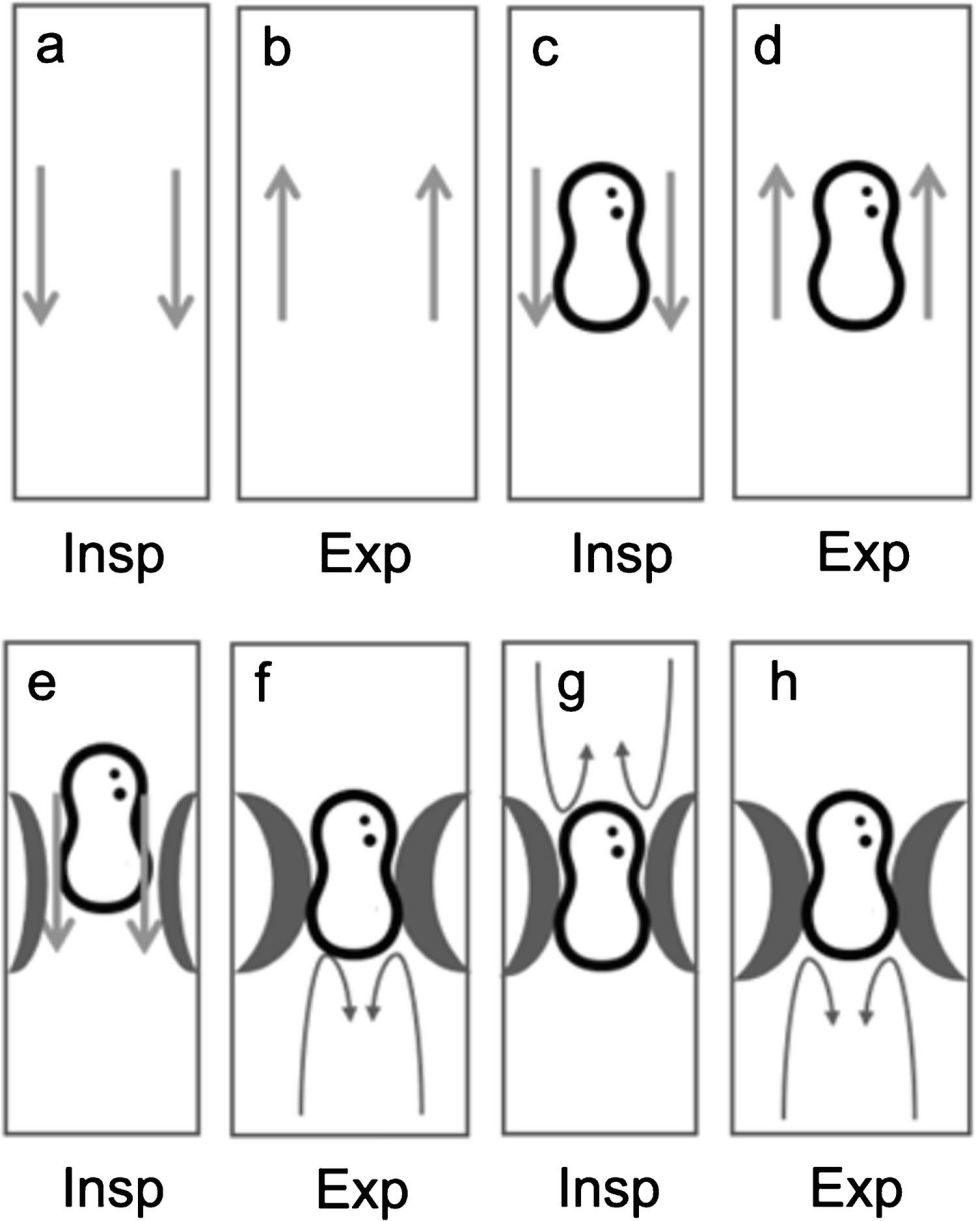
**a**, **b** Normal inspiration (**a**) and expiration (**b**) in the absence of a foreign body. **c**, **d** Normal inspiration (**c**) and expiration (**d**) in the presence of an airway foreign body. **e**, **f** A foreign body that permits air to pass by on inspiration (**e**) but is trapped during expiration (**f**) causing obstructive emphysema. **g**, **h** Complete obstruction during inspiration (**g**) and expiration (**h**)

**Fig. 3:**
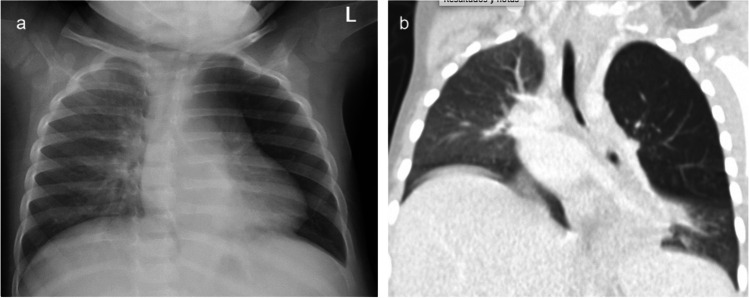
Radiographs of an 18-month-old girl. **a** The anteroposterior (AP) view indicates the left lung appears more radiolucent than the right, with a slight diminution in lung markings. There is a suspicion of a foreign body in the left main bronchus causing a so-called interrupted bronchus sign. **b** Coronal view computed tomography (CT) with contrast, in lung window, confirms the presence of an inhaled foreign object in the left main bronchus, which has resulted in air trapping in the left upper lobe

The specific procedure may vary, but most hospitals perform chest radiographs, frontal posteroanterior radiographs in older children and anteroposterior radiographs in younger children during full inspiration and forced expiration [6]. Some centres also include lateral decubitus views in children who do not comply with instructions (Fig. 1) [7]. The decubitus views are particularly useful if the child is uncooperative in having radiographs taken during full inspiration and forced expiration. As most foreign bodies are radiolucent, aspirated foreign bodies often manifest through indirect findings on a radiograph. Key findings to look for include hyperinflation or obstructive emphysema, mediastinal shift, pneumonia, atelectasis and complete lung opacification. All of the aforementioned radiographic findings are dependent on the size and location of the aspirated foreign body. The size of the foreign body determines the extent of its effect, as well as the type of biomechanical impact it has on the lungs and airways (Fig. 2). The most common version of the foreign body is the so-called ball-valve mechanism. The “ball-valve mechanism” means that air can pass by the foreign body during inspiration but is trapped in the distal lung on expiration which causes air trapping which is seen as hyperlucent lung parenchyma on a chest radiograph (Fig. 3). Mediastinal shift is a secondary indicator when the hyperinflated lung expands sufficiently to displace the mediastinum to the opposite side. Occasionally, the foreign body may occlude the entire bronchus and induce atelectasis in a manner similar to a mucus plug. The extent of the atelectasis depends on the location of the foreign body, ranging from a subsegmental level to complete atelectasis of a lung if it obstructs the main bronchus.

The radiograph itself displays low sensitivity and specificity, in addition to being contingent on the expertise of the individual evaluating the radiographic images. The radiologist’s role is to report the presence of a detectable radiopaque/radiolucent foreign body, the nature of the object (if possible and particularly if suspected to be a battery, sharp object or magnet) and any signs of perforation. The sensitivity in detecting foreign body aspiration has been reported to range between 60 and 85%, with a specificity of 32% to 68% [8–10]. The low sensitivity and specificity have prompted some studies to suggest that bronchoscopy should be performed on all patients with a history of choking, even in the presence of minimal symptoms and normal radiological findings [5].

## Fluoroscopy

Fluoroscopy of the airways can be employed as a dynamic examination to visualise if there is a displacement of the mediastinum and diaphragm. It can contribute to the diagnosis in most cases of bronchial foreign body but is less effective when the foreign body is situated in the laryngotracheal region [11]. The breathing movement ought to be symmetrical, and real-time assessment heightens the probability of detecting asymmetry in the breathing motion and unilateral air trapping. The hilum shifts towards the obstructed lung during inspiration, and during expiration, it moves in the opposite direction, towards the remainder of the lung, which forces the air out. This movement is known as the Holzknecht-Jacobson sign and indicates obstruction of one of the main bronchi [12]. Imaging the patient in the lateral decubitus position, lying on the suspected affected side, can further reveal that the occluded lung remains unaltered in volume and immobile during respiration.

## Computed tomography

Computed tomography (CT) can prove advantageous in challenging cases, as it provides a more three-dimensional perspective of the foreign body and its positioning. It should also be considered in patients with recurrent pneumonia on the same side, to rule out foreign bodies as a cause of the pneumonia. The advantage of CT is that it markedly enhances the sensitivity in comparison to radiograph and fluoroscopy; however, all radiological examinations must be medically justified, adhere to the ALARA (as low as reasonably achievable) principle and follow the Image Gently guidelines (www.imagegently.org). From a paediatric radiology standpoint, it is imperative that we do not increase the amount of ionising radiation for this patient cohort.

Very low-dose CT has become a feasible alternative, as technological developments have facilitated reduced acquisition time and decreased radiation dosage [13]. With the assistance of a physicist, the radiation dosage can be reduced to be equivalent to or lower than a combination of chest radiograph and fluoroscopy [14, 15].

**Fig. 4:**
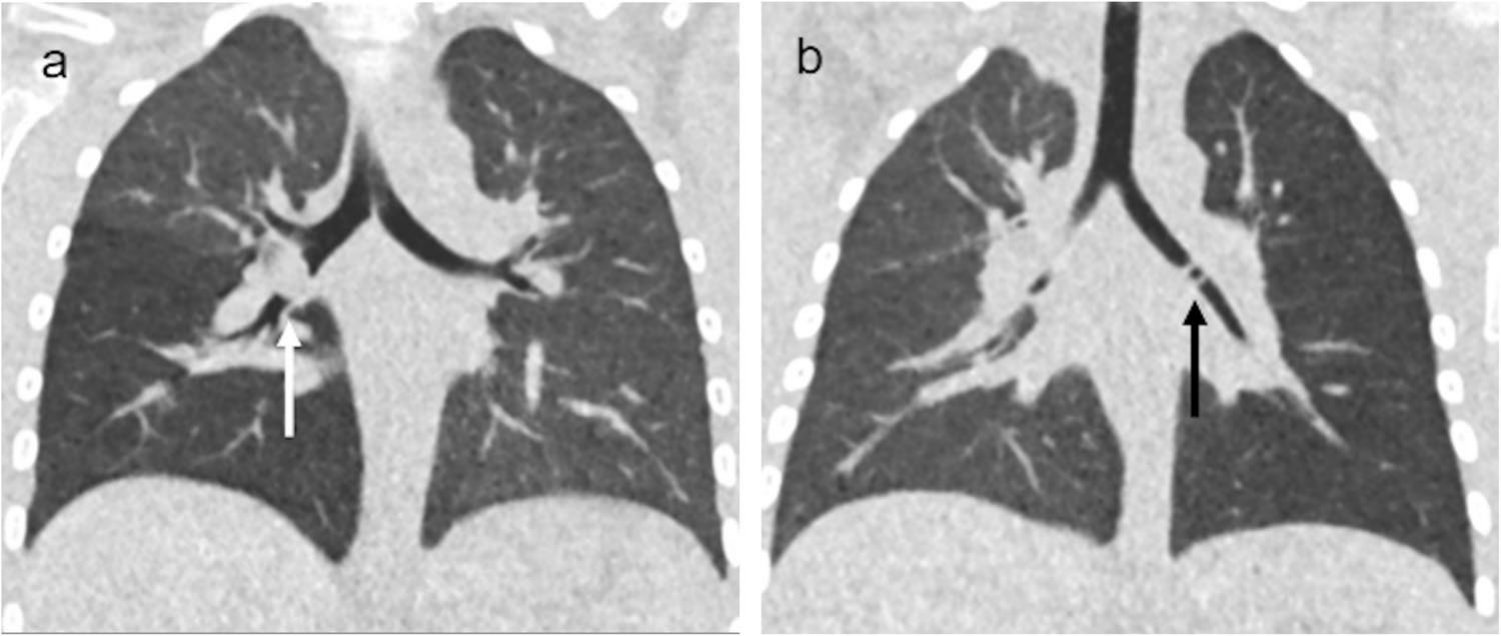
**a** Coronal view computed tomography (CT) without contrast, in lung window, of a 7-year-old girl with a piece of carrot in the right bronchus (*white arrow*). **b** Displays a coronal view CT without contrast, in lung window, of a 2-year-old with a Perler bead in the left main bronchus (*black arrow*). Both examinations had an effective dose of 0.04 mSv. Republished from *Diagnosis of a foreign body aspiration with ultralow-dose CT using a tin filter: a comparison study,* Courtesy of Dr Lena Gordon Murkes [14]

A comparative study showed that very low-dose CT with a mean effective dose of 0.04 ± 0.12 mSv showed that the sensitivity increased from 33% (7–70%) for the combination of radiograph and fluoroscopy to 100% (66–100%) for very low-dose CT with unchanged specificity between 96–98% [14]. The study concluded that CT could diagnose solid objects as well as hollow objects, like Perler beads (Fig. 4). Other studies have reported that low-dose CT and virtual bronchoscopy are non-invasive radiological modalities that can be used easily in the investigation of foreign bodies of the airway in children. CT and virtual bronchoscopy provide the exact location of the obstructive pathology prior to bronchoscopy [16]. This contradicts other studies that found vegetable fragments can be missed on CT [17]. It is likely that future technological advances and the emergence of photon counting CT will improve image quality at the same or even lower mean effective dose [18].

It has been suggested that ultra-low-dose CT may be employed as a primary diagnostic tool if readily available, and the dose is equivalent to or lower than a chest radiograph and fluoroscopy combination [14, 16]. The primary advantage is that this will diminish the risk of misdiagnosis and unnecessary bronchoscopy, which are expensive and carry additional risk for the patient.

## Ultrasound

Ultrasound currently plays a supportive yet limited role in detecting foreign bodies in the airways and gastrointestinal tract. While it is not the primary imaging modality for these regions, ultrasound can be useful in specific cases, particularly for identifying radiolucent objects such as plastic or wood, which may not be visible on radiographs [19]. Sonographic diagnosis of a foreign body depends on both primary and secondary signs. The foreign object itself typically appears as an echogenic mass with a dense acoustic shadow (primary signs). Secondary signs, such as tissue oedema or haemorrhage, present as a hypoechoic area surrounding the foreign body [20]. The movement of the diaphragm can be monitored via ultrasound. Reduced movement of the diaphragm may be caused by injury, such as diaphragmatic paraesthesia or a foreign body obstructing the main bronchus [21].

In the gastrointestinal tract, ultrasound can assist in detecting foreign bodies that cause obstruction or have migrated outside the intestinal lumen, potentially leading to complications like abscesses. It has been proposed that ultrasonography provides comprehensive information about the size, structure and location of abdominal foreign bodies, including their depth and relationship to surrounding organs [22]. These aforementioned case reports support the growing evidence that skilled use of ultrasonography, a non-invasive and painless diagnostic tool, can effectively identify abdominal foreign bodies. Additionally, ultrasound can be a non-ionising method to follow the known foreign bodies’ passage through the gastrointestinal tract. However, ultrasound is generally less effective for detecting foreign bodies in the airways, where imaging techniques such as radiographs or CT scans are typically preferred.

## Ingested foreign bodies

There are numerous locations where ingested foreign bodies can cause obstructions. Within the oesophagus, there are three common locations where obstructions may occur: at the level of the superior oesophageal sphincter, at the level of the aortic arch, and at the level of the inferior oesophageal sphincter [23, 24]. The removal of foreign bodies in the oesophagus is considered mandatory, including impacted food. Other anatomical locations where foreign bodies can cause obstructions include the pyloric sphincter and ileocaecal valve (especially large objects), as well as the rectosigmoid colon and anus. An object with a diameter larger than 25 mm is unlikely to pass through the pylorus [25]. Objects that are longer than 6 cm have a difficult time to pass through the duodenum and are also unlikely to pass through the ileocaecal valve [26]. Further areas prone to obstruction are locations with acquired or congenital strictures, such as anastomosis following atresia surgery. If a radiograph is performed, it needs to include the entire gastrointestinal tract, i.e. neck, chest and abdomen. One can determine if a flat circular foreign body, such as a coin or button battery, is situated in the oesophagus or trachea depending on its appearance. A circular foreign body appears round in the anterior–posterior view if situated in the oesophagus, and narrow if located in the trachea, as the posterior wall lacks cartilage rings and thus is not rigid. The image is reversed in a lateral view. Additionally, one ought to detect the trachea on the lateral view to ascertain if the foreign body resides within the trachea or posterior to it. The management of an ingested foreign body depends not solely on its location, but also on the type of foreign body ingested (Table 1). A clinician ought always to consider potential poisoning from ingested substances, whether deliberate or accidental. Radiographic follow-up can detect if an injury has occurred and early indications of it being retained [27]. If there is suspicion of perforation, then CT should be considered. Furthermore, ultrasound may be considered to evaluate the progression of a foreign body in the abdomen or if it has caused secondary effects or complications.

**Table 1:** Suggested radiographic follow-up depending on the nature of the object and its location within the gastrointestinal tract

Type of object	Radiographic follow-up
Coins	Radiographic follow-up once a week is sufficient, unless the patient is symptomatic [28]
Batteries	Urgent removal if situated in the oesophagus. If in the stomach and beyond, follow-up after 3–4 days to monitor passage; batteries remaining in the stomach longer than 48 h should be retrieved endoscopically [25]
Magnets	Serial radiographs are advised if the object continues to show mobility and the patient remains asymptomatic [29]
Sharp objects	If the sharp object has passed the duodenum, it should be monitored daily to document its passage [25]

### Coins

Coins are one of the most commonly ingested foreign objects, and patients typically present with dysphagia. Coins can cause mechanical obstruction, but are considered relatively safe as they lack sharp edges. One consideration is whether the metal composition of the coin could potentially have a toxic effect. The US penny has a high zinc content (97.5% zinc, 2.5% copper) and the Euro 10c, 20c and 50c coins consist of 89% copper [30, 31]. All metal coins risk reacting with the gastric acid in the stomach. The free metal ions can then have local or systemic effects on an individual. It has been reported that coins in hydrochloric acid lose between 0.43–11.3% of their weight over the course of a week [32]. The dissolved ions can then be absorbed or react further with the hydrochloric acid and become a salt. Zinc can react with gastric acid and result in zinc chloride. Zinc chloride causes vomiting, gastritis and, secondarily to this, gastro-oesophageal burns and haemorrhage that can ultimately result in scarring. Gastric acid can enable the body to absorb copper, which can cause acute liver failure. Treatment of ingested coins generally depends on their location and the symptoms. Urgent removal of any foreign body is indicated if the obstruction causes an inability to manage oral secretions. A coin that fails to pass the pylorus after 48 h may indicate the need for endoscopic removal, but this can vary depending on local practice [24]. The main role of the radiologist is to identify whether the foreign body is a coin, where it is located, and if it causes any obstruction.

### Batteries

**Fig. 5:**
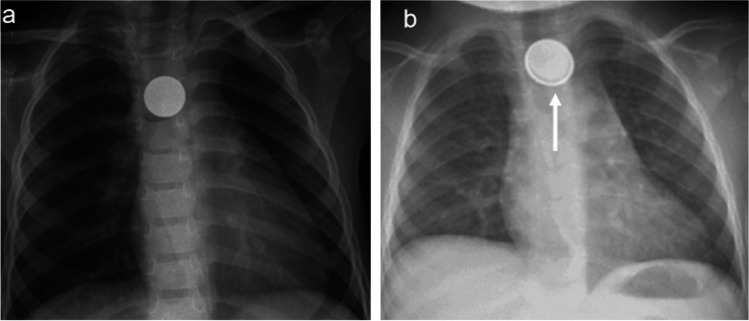
Lung radiographs, anteroposterior (AP) views of foreign bodies in the mediastinum. **a** A coin which appears as a solid structure in a 3-year-old boy. **b** A button battery with a visible double ring (*arrow*) which is pathognomonic, in a 2-year-old girl

**Fig. 6:**
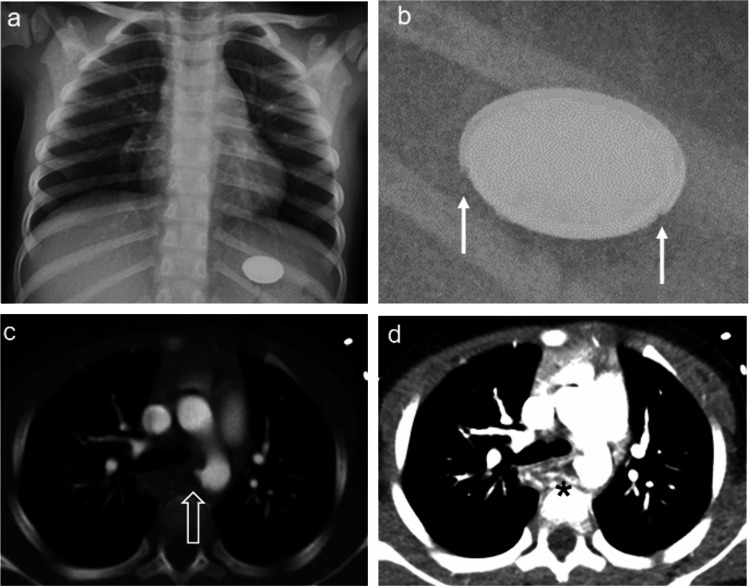
A 2-year-old girl presenting with haematemesis. **a** An anteroposterior radiograph displays a button battery within the stomach. **b** Magnified view of (**a**) shows small erosions (*solid white arrows*). **c** Axial view computed tomography (CT) with contrast, in soft tissue window, shows a pseudoaneurysm from the aortic arch, adjacent to the oesophagus (*open white arrow*). **d** Axial view CT with contrast, in soft tissue window, shows fluid accumulation between the oesophagus and the pseudoaneurysm, which was confirmed to be necrosis caused by the button battery (*asterisk*)

Ingested batteries require more direct management, particularly button batteries, which can sometimes be mistaken for coins (Fig. 5). A coin appears solid on a radiograph, whereas a button battery has a metal casing that gives it a double-ring appearance. An irregular contour of the double ring is an indication that the encasing is no longer intact, and potential leakage of battery acid should be considered. Batteries cause an external current that hydrolyses fluids in tissues and creates hydroxide ions (OH-) at the negative pole of the battery. The battery itself causes physical pressure, and its contents, especially battery acid, can cause coagulation necrosis [33]. The harmful effect on the soft tissues is most prominent in the oesophagus, and any detected batteries in the oesophagus should be extracted as soon as possible [34]. Battery leakage has been documented as early as 2 h after being in a solution with the same pH as gastric acid [35]. It is therefore crucial for the radiologist to detect the presence and location of a battery, especially in the oesophagus, so it can be extracted before permanent damage occurs (Fig. 6).

### Magnets

**Fig. 7:**
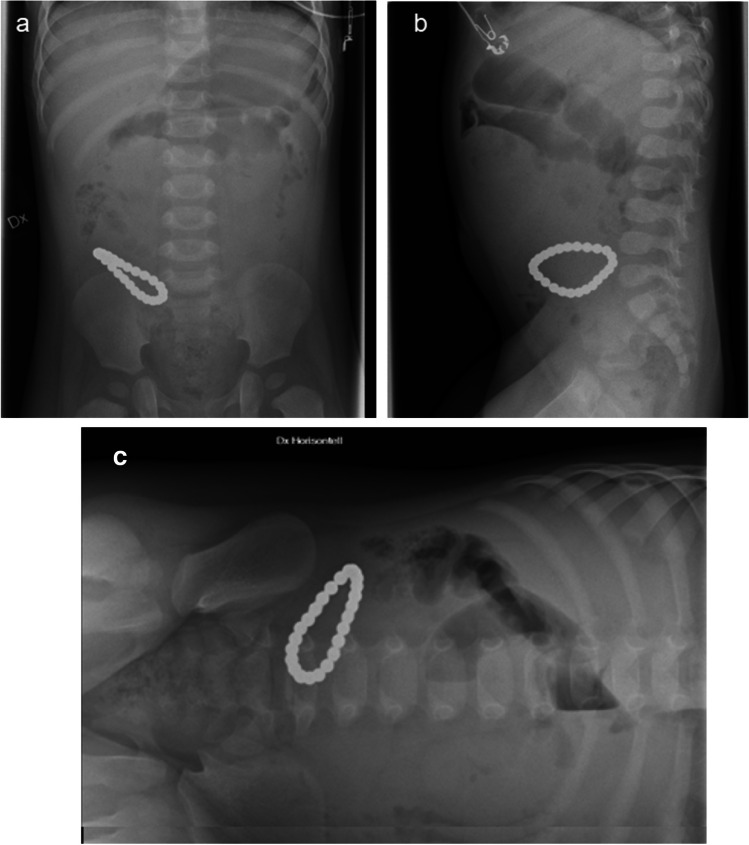
Anteroposterior (**a**), lateral (**b**) and left lateral decubitus (**c**) abdominal radiographs of a 1-year-old boy who presented at the emergency department with vomiting and a clinical suspicion of ileus or gastroenteritis. The radiographs reveal a radiopaque pearl bracelet in the bowel. The boy’s guardian denied known ingestion of foreign bodies

Ingestion of magnetic objects is common and can have potentially dire consequences. Magnets can be found in toys for children under the age of four, in building blocks, in wooden games, and in more traditional toy cars or trains. Magnets can be of various shapes and sizes with a metallic opacity on a radiograph. Magnets in different bowel loops can become attracted to one another through the bowel wall. Clinical information about the suspected foreign body is critical to making a correct diagnosis, but if there are two or more metallic objects adjacent to one another, it should raise concern for multiple ingested magnets (Fig. 7).

**Fig. 8:**
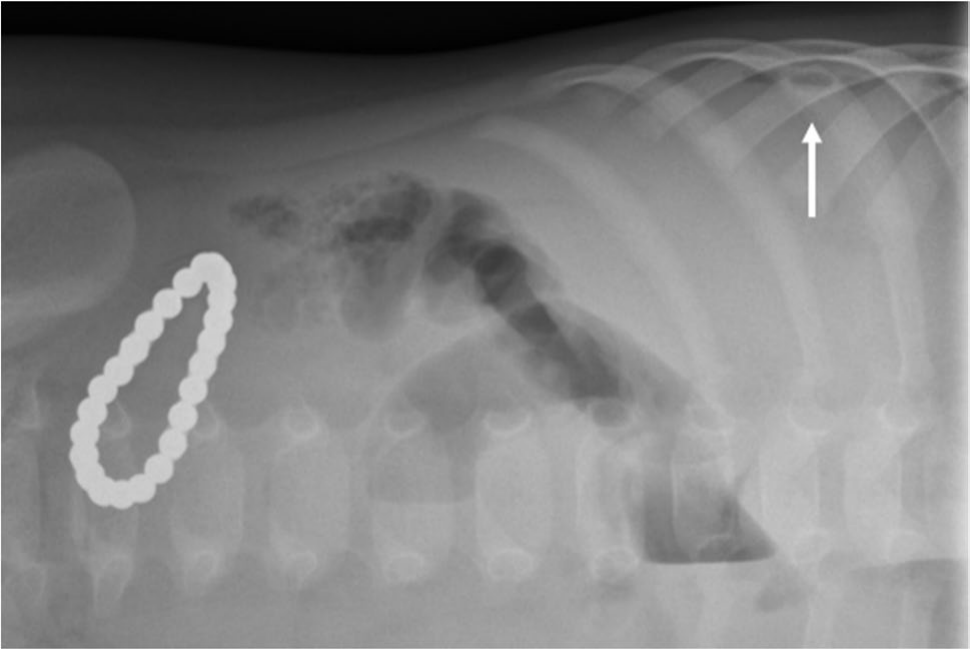
Magnification of the right decubitus view radiograph (1-year-old boy) shows a small quantity of free air between the liver and the diaphragm (*arrow*). Surgery verified perforation of four small bowel loops and 25 small magnetic balls

A magnetic object does not need any intervention if it is a single magnet without the presence of other metallic objects. However, if more than two magnets (or a magnet + magnetic object) are ingested, urgent intervention is indicated if they can be reached by endoscopy or if there are signs of perforation of the bowel caused by pressure from the magnets (Fig. 8). Bowel necrosis caused by magnets can result in entero-enteric fistula [36].

A magnet looks like any metallic object and can therefore be difficult to identify without information about the suspected type of foreign body. The radiologist should always raise concerns if two or more magnets or metallic objects are seen in close relation to one another.

### Sharp objects

Sharp objects can occasionally be ingested by children and older persons with self-destructive conduct. Ingested sharp objects, from razor blades to pins and fish bones, can cause perforation of the hollow viscera. A potential complication of sharp objects is that they may migrate out of the airways or gastrointestinal tract and cause local complications ranging from subcutaneous abscess of the neck or mesenteric fat to being present in the liver [37–39]. The location of the sharp object and any signs of perforation and migration determine the necessary management. The majority of ingested foreign bodies (80–90%) pass through the gastrointestinal tract spontaneously, and only 1% necessitate surgery [40]. Endoscopy is the preferred method if the object is located oral to the pylorus.

## Conclusion

Radiology plays a significant role in diagnosing foreign bodies in the airway and/or the gastrointestinal tract. Symptoms can be non-specific, and most foreign bodies in the airways are radiolucent. One-sided hyperinflation, mediastinal shift and one-sided recurrent pneumonia are indicators that may suggest an aspirated foreign body in the airway. A suspected ingested foreign body in the gastrointestinal tract necessitates examination of the entire gastrointestinal tract, i.e. neck, chest and abdomen. Button batteries exhibit a characteristic double-ring appearance on a radiograph and may be readily diagnosed. A prompt and accurate diagnosis is essential to determine the correct treatment, as foreign bodies can cause chemical damage and/or perforation (in the case of batteries and magnets) that can potentially be fatal.

## Data Availability

No datasets were generated or analysed during the current study.
